# Effect of Charge Distribution Along Anionic Polyacrylamide Chains on Quartz Adsorption: A Molecular Dynamics Study

**DOI:** 10.3390/polym18030414

**Published:** 2026-02-05

**Authors:** Gonzalo R. Quezada, Karien I. García, Enoque Diniz Mathe, Williams Leiva, Eder Piceros, Pedro Robles, Ricardo I. Jeldres

**Affiliations:** 1 Departamento de Ingeniería de Procesos y Bioproductos, Facultad de Ingeniería, Universidad del Bío-Bío, Concepción 4030000, Chile; 2 Facultad de Ingeniería, Universidad del Bío-Bío, Concepción 4030000, Chile; kgarcia@ubiobio.cl; 3 Departamento de Ingeniería Química y Procesos de Minerales, Facultad de Ingeniería, Universidad de Antofagasta, Antofagasta 1240000, Chile; enoque.diniz.mate@ua.cl (E.D.M.); ricardo.jeldres@uantof.cl (R.I.J.); 4 Facultad de Ingeniería, Universidad San Sebastián, Sede Concepción, Concepción 4030000, Chile; williams.leiva@uss.cl; 5 Facultad de Ingeniería y Arquitectura, Universidad Arturo Prat, Iquique 1110939, Chile; edpicero@unap.cl; 6 Escuela de Ingeniería Química, Pontificia Universidad Católica de Valparaíso, Valparaíso 2340000, Chile; pedro.robles@pucv.cl; 7 Advanced Mining Technology Center (AMTC), Universidad de Antofagasta, Antofagasta 1240000, Chile

**Keywords:** polyacrylamide, quartz, molecular dynamics, polymer design, polymer adsorption

## Abstract

The interfacial behavior of polyelectrolytic flocculants is governed not only by their chemical composition but also by the molecular-scale distribution of charged and neutral segments, which directly influences transport, adsorption, and interfacial stability. In this work, classical molecular dynamics simulations are used to elucidate how charge-site architecture controls the conformation, dynamics, and adsorption stability of anionic polyacrylamides at the quartz–water interface. Polymer architectures ranging from homogeneous charge distributions to block-like arrangements were systematically analyzed at constant molecular weight and global charge density. The results show that increasing charge segregation induces more compact conformations, enhanced translational mobility in solution, and reduced solvent accessibility. At the interface, polymers containing extended neutral blocks exhibit significantly more stable adsorption on quartz than polymers with homogeneously distributed charges, consistent with the low surface charge density of silica. These findings demonstrate that charge-site distribution is an independent and critical design parameter governing polymer–surface interactions. From a chemical engineering perspective, the results provide fundamental insight relevant to the rational design of polymeric additives for solid–liquid separation, flocculation, and sustainable mineral processing applications.

## 1. Introduction

Mining in Chile is an essential pillar of its economic development and identity, led by the production of copper and complemented by the exploitation of lithium, iron, gold and silver [1,2,3]. However, much of its operations are located in arid and environmentally fragile areas, facing challenges such as water scarcity, tailings management, and the need for more sustainable processing technologies [4,5]. In this context, solid–liquid separation during tailings thickening is critical for water recovery and system stability. This process seeks to settle fine particles suspended in aqueous pulps, the efficiency of which depends on the interactions between the particles and chemical additives [6]. Flocculants and coagulants are essential: the former generate polymeric bridges that agglomerate particles, while the latter neutralize surface charges and promote their coalescence [7]. Various studies have shown that the combination or modification of additives significantly improves sedimentation rate and effluent clarity. The simultaneous use of polymers produces synergistic effects [8], while flocculants with aromatic rings or modified structures allow for greater removal of contaminants [9]. Even optimizations in thickening tank design can significantly reduce operating times [10]. Together, these studies show that the chemical formulation and the process conditions determine the efficiency of thickening [11]. At the same time, the search for more sustainable alternatives has driven the development of naturally occurring or structurally modified flocculants. Among them, hydrophobically modified chitosan (TRC) has shown strong aggregation capacity thanks to the combination of polar and nonpolar domains [12]. Likewise, branched polymers or mixtures of linear and branched flocculants have shown different behaviors depending on their configuration and the physicochemical properties of the medium [13,14,15,16,17,18,19,20]. From this set of results, it can be deduced that the macromolecular architecture of the flocculant, beyond its chemical composition, controls the shape, resistance and stability of the flocs, which opens the way to the rational design of polymers adapted to specific conditions of pH, salinity and surface load.

At a fundamental level, the efficiency of flocculation is explained by the adsorption of water-soluble polymers derived from acrylamide or acrylate on the mineral surfaces of the tailings. This process depends on the quantity and configuration of the adsorbed chains, which determine mechanisms such as bridging, patching, or floc restructuring under shear [21,22,23]. The polymer’s architecture (random, block, graft), its molecular weight, and its charge density control the arrangement of the chains on the surface, generating adsorbed regions (“trains”) and extended regions (“loops” and “tails”). These characteristics, in turn, influence floc compaction and sedimentation rate [23]. The predominant minerals in the tailings—quartz, montmorillonite, kaolinite, and pyrophyllite—possess abundant silanol (Si–OH) and aluminol (Al–OH) groups, giving them high reactivity to charged polymers [23,24,25]. Classical experimental studies, such as those by Heller and Keren [24], showed that the adsorption of anionic PAM (PAM90) on pyrophyllite is higher at moderate pH (≈6) and in saline media than in pure water, which shows that an intermediate ionic strength favors the compaction of the chains without inducing their desorption. On the other hand, in sodium montmorillonite, a polymer with a lower degree of hydrolysis (ACC86) showed a higher adsorption, which shows the determining role of aluminol groups. For their part, Lee and Schlautman [22], reported that the adsorption of nonionic PAM on kaolinite increases with molecular weight, although excessive molecular weights reduce effective contact due to chain entanglement. From this set of works it can be deduced that adsorption does not depend only on the total charge of the polymer, but also on its conformational distribution, which is modulated by pH, salinity and mineral composition. These factors govern the balance between extended chains (which facilitate bridging) and compact chains (which favor local neutralization).

Advances in molecular dynamics (MD) simulations have made it possible to directly visualize the interactions between polymers and mineral surfaces. Yang et al. [26] demonstrated that the adsorption of polyacrylamide on quartz occurs primarily by hydrogen bonding between the amide groups and the silanol groups. They also observed that monomers with acid groups (AA, AMPS) have a higher affinity than neutral ones (AM), which confirms that the density and accessibility of the charges determine the intensity of adsorption. Quezada et al. [27] extended this analysis, showing that the adsorption of HPAM on quartz decreases in pure water and at low surface loads, but intensifies under saline conditions, where counterions stabilize the adsorbed chains. These simulations allow us to conclude that the adsorption process is competitive between the polymer and the water molecules due to the active sites on the surface. The presence of multivalent salts or cations modifies this competence and alters the morphology of the adsorbed layer. In other words, the simulations have confirmed what was previously inferred empirically: the electrostatic environment of the system controls the way the polymer anchors and rearranges itself on the surface.

Subsequent research extended this framework to more complex polymers. Random and graft copolymers have made it possible to adjust the adsorption properties by incorporating hydrophilic or hydrophobic blocks. Bijsterbosch et al. [28] observed that polyacrylamide graft copolymers with polyethylene glycol (PAAm–PEO) are more adsorbed on silica than on titania, due to the formation of extensive loops that maximize surface contact. In contrast, random anionic copolymers of acrylamide–acrylic acid showed selectivity for mixed surfaces of SiO_2_–TiO_2_ according to the relative proportion of oxides [29]. Studies on block copolymers, such as PEO–PPO–PEO triblocks, demonstrated the ability to form organized “trains, loops, and tails” structures, in which the PPO block acts as a hydrophobic anchor and the PEO block stabilizes the surface layer [30,31,32]. Research with cationic or zwitterionic copolymers [33,34,35] and with functionalized graft-from grafts on silica [36,37,38,39,40], showed that the molecular configuration and the degree of graft determine both colloidal stability and resistance to desorption. From this literature, it can be deduced that adsorption behavior is not universal but depends on the balance between chemical affinity and conformational restriction of the polymer. Therefore, architecture (and not only chemistry) defines adsorption efficiency and the formation of stable functional layers on silica and clays.

Despite extensive experimental and computational research on polymer adsorption and flocculation, most studies focus on varying molecular weight or overall charge density, implicitly assuming that polymers with identical global composition behave equivalently. However, from a fundamental engineering science standpoint, the intramolecular sequence and spatial distribution of charged groups may critically alter polymer conformation, transport properties, and interfacial organization, even at fixed global charge density. This work addresses this gap by using molecular dynamics simulations to isolate the effect of charge-site distribution along anionic polyacrylamide chains interacting with a quartz–water interface. By systematically varying charge architecture while keeping molecular weight and total charge constant, we aim to establish structure–property–interaction relationships that connect molecular design to interfacial behavior. Such understanding is essential for advancing predictive models and rational design strategies in polymer-assisted solid–liquid separation processes and related chemical engineering applications.

## 2. Materials and Methods

### 2.1. Compounds

In these simulations, the acrylamide polymer was defined, as it had already been evaluated in previous work [41]. To do this, we first set a polymer size necessary to be able to study conformations in addition to adsorption on mineral surfaces. Therefore, a polymer comprising 80 monomers was employed, which is larger than those considered in previous studies. This increased chain length allows for a more realistic representation of polymer conformations, intramolecular flexibility, and surface–polymer interactions, albeit at the expense of larger simulation domains and higher computational cost. With the charge density set at 25%, we then have 20 charged monomers. Table 1 presents the configurations studied in this work. Configuration 1 is the homogeneous one, in which the charged groups are equidistant from each other along the chain. As the N increases, the charged groups clump together, generating these new polymer configurations, keeping the charge density constant.

The quartz surface was generated by a quartz supercell of 32 × 10 × 3, with the surface exposed (101), according to previous work [42] using an in-house script developed by the authors where surface dimension and degree of deprotonation can be imposed. It has an area of ~200 nm^2^, enough to interact with the 80-monomer polymer. The system is simulated under pH 8 conditions, in which 40 deprotonated siloxanes are fixed to generate a charge of around −0.03 C/m^2^. Finally, the counterions were simulated as Lennard-Jones type spheres and the water was modeled using the 3-site model, with two hydroxyl groups and one oxygen group.

### 2.2. Force Field

To model the polymer, the parameters already described in previous works [41] are used, which uses the GAFF and with charge adjustment by means of the RED algorithm that is downloaded in RESP. In the case of quartz, the force field was based on CLAYFF, with modifications of the surface charge due to deprotonation. Surface deprotonation and charge assignment for quartz were implemented following the protocol of Koutril et al. [43]. The resulting partial charges for surface atoms and deprotonated silanol groups are explicitly defined in the topology files provided in the Supplementary Information. The ions were modeled with the IOD force field of Li et al. [44] and water with the SPC/E model [45].

### 2.3. Initial Setup

**Figure 1:**
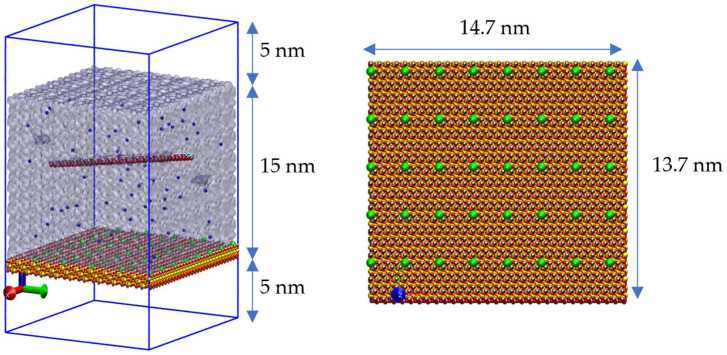
Initial molecular dynamics configuration. Deprotonated sites on the quartz (101) surface (green spheres) correspond to a surface charge density of −0.03 C m^−2^. Water is shown as a blue continuum, while Na^+^ (blue) and Cl^−^ (cyan) ions are explicitly represented. The polymer chain is initially placed at the center of the simulation box along the z-axis, equidistant from the vacuum region and the mineral surface.

To analyze the effects of the charged group configuration, two systems were generated. The first consists of a classic cubic box system with 15 nm sides. The polymers under study are positioned within the box along with their counterions to neutralize it. In this case, sodium is used because this type of polymer is stabilized and commercially available as a sodium salt of anionic flocculants. The second system consists of a box of similar dimensions but adapted to allow the quartz crystal to generate a periodic configuration. Therefore, the dimensions were 14.7 × 13.7 × 15.0 nm. A 10 nm vacuum was added along the *z*-axis (25 nm total along the *z*-axis) to minimize the polymer’s interaction with the periodic images in that direction and focus its interaction only with the exposed surface. The polymer is placed at a distance greater than 5 nm from the surface to monitor the adsorption evolution during the simulation. A schematic representation of the initial simulation box, including the coordinate system, quartz slab, vacuum region along the z-axis, and initial polymer placement, is provided for clarity (Figure 1).

### 2.4. Molecular Dynamics

The simulations were performed using Gromacs software, version 2022.1 [46], installed with support for OpenMP, GPU, and AVX2_256 SIMD instructions. The simulation procedure differs between the two systems in Figure 1; a flow diagram is presented for each. In System S1, a traditional simulation is performed in which the minimization and equilibration steps prepare the system to resemble a realistic configuration. The annealing method is used to favor the most stable polymer conformation. In System S2, a similar scheme is initially applied, but in step 3, the ions are adjusted, leaving the polymer constrained; this serves to form the DLVO layers on the quartz surface. Then, in step 4, a steered MD (SMD) approach is used, in which the polymer is guided to adsorb in three different configurations: two in which the polymer interacts with one of its ends and one in which it interacts with the center of its chain. Then, in the final step, the system is released to verify whether this interaction is stable, whether it adopts another, or whether it desorbs completely. The simulations were performed with 3 repetitions to increase the sampling rate.

**Figure 2:**
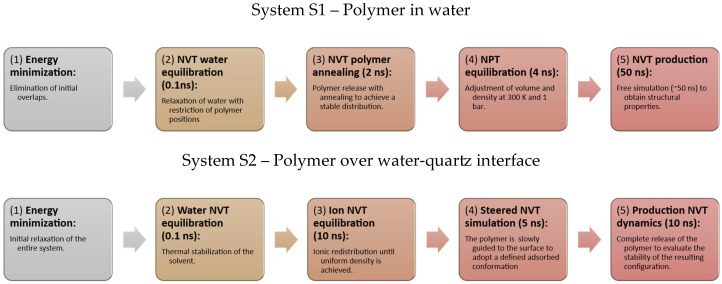
Flow of simulations performed for the two main systems studied.

**Figure 3:**
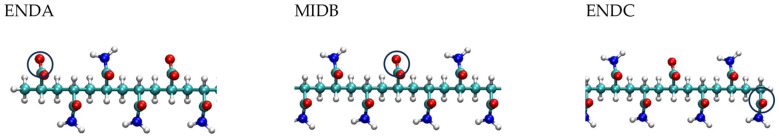
Choice of the oxygen atom (black circle) used as a pull point in the steered molecular dynamics (SMD) simulations for the ID = 1 case, corresponding to the ENDA, MIDB and ENDC configurations (start, middle and end of the chain).

In the SMD step of System S2 (Figure 2), the polymer was guided to the surface using an oxygen atom belonging to a polymer functional group as the pull point. To evaluate the influence of position along the chain, three reference configurations were defined: ENDA, MIDB, and ENDC, corresponding to an oxygen atom located at the leading, middle, and trailing ends of the chain, respectively. Figure 3 shows an illustrative example for the ID = 1 case, where the selected atom in each configuration is indicated by a circle. For systems with ID > 1, the same selection criteria were maintained, always using the same oxygen atom associated with functional groups located at the ends and in the middle region of the chain, thus ensuring a consistent comparison between configurations. In the specific case of ID = 20, the MIDB configuration corresponds to an oxygen atom belonging to an acrylamide group, because the chain rearrangement causes the charged groups to preferentially concentrate at the ENDA end. This choice allows the geometric definition of the polymer’s mid-region to be preserved, even though the charge distribution differs from systems with lower ID.

All molecular dynamics simulations were performed using a timestep of 2 fs during production runs, while shorter timesteps (1 fs and 0.5 fs) were employed during equilibration and energy minimization, respectively. Temperature was maintained at 300 K using the V-rescale thermostat during initial equilibration stages and the Nose–Hoover thermostat during production and SMD simulations. Pressure coupling (Parrinello–Rahman, 1 bar) was applied only during the NPT equilibration of System S1. For the polymer–quartz system (System S2), after equilibration, steered molecular dynamics (SMD) were performed to guide the polymer toward the surface along the z-direction, using an oxygen atom from the polymer backbone as the pulling reference. Three pulling configurations (ENDA, MIDB, and ENDC) were considered. Subsequently, unconstrained production runs of 5 ns were carried out to assess adsorption stability. All simulations were performed in triplicate. Complete topology files, force field parameters, and molecular dynamics input files are provided in the Supplementary Information.

### 2.5. Processing

For trajectory analysis, the tools incorporated in GROMACS were used to describe the structural and dynamic evolution of the polymer. The gmx gyrate command was used to calculate the radius of gyration and evaluate changes in chain compaction. The mobility of the polymer and solvent was determined with gmx msd by calculating the root mean square displacement. Additionally, gmx sasa was applied to estimate the surface area accessible to the solvent, a relevant indicator of the polymer’s exposure to the medium. Finally, gmx mindist was used to identify the minimum distances between the polymer and the mineral surfaces, allowing for the detection of approach and adsorption events. The data generated by these tools were subsequently organized, analyzed, and plotted using Python (3.12.12) scripts and libraries such as NumPy (v2.0.2), Pandas (v2.2.2), and Matplotlib (v.3.10.0), enabling a systematic comparison of the different simulated conditions.

## 3. Results

### 3.1. Conformation and Diffusion in Water

**Figure 4:**
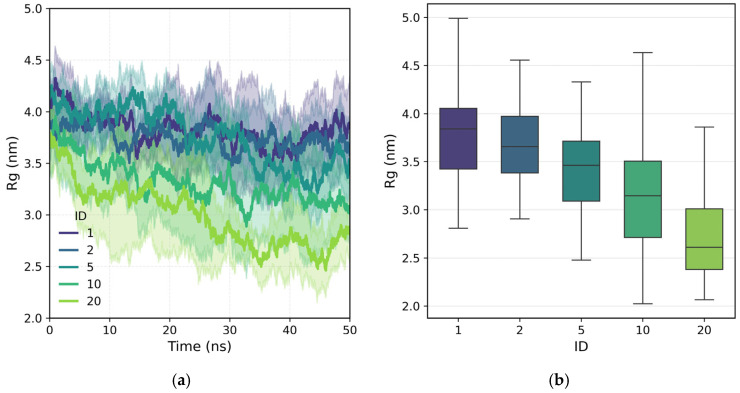
Radius of gyration (Rg) of the studied polymers in aqueous solution. (**a**) Temporal evolution of Rg and (**b**) distribution of the Rg values over the last 20 ns.

Figure 4 presents the temporal evolution and distribution of the radius of gyration (Rg) of polymers with different configurations of their charged groups. The results show a systematic decrease in Rg as the charges cluster, indicating a transition from extended conformations to more compact structures. In polymers with homogeneously distributed charges, the average radius of gyration remains close to 4 nm, while in configurations with longer charged blocks (10–20 nm), it decreases to approximately 2.5–3 nm. This contraction is associated with the increase in the continuous neutral segment of polyacrylamide (PAM), which tends to curl in the absence of internal electrostatic repulsions. Since the total charge density remains constant, the observed differences are attributed solely to the charge microarchitecture. In low-ionic-strength conditions, where only polymer counterions are present, repulsions between charged monomers are the main factor promoting extended conformations, while local neutrality favors partial collapse. These results demonstrate that the spatial organization of charges strongly influences the polymer’s conformation, even in pure water, which anticipates different behavior in ionic media or in the presence of mineral surfaces. It must be noted that all polymer architectures share the same backbone length, with a fully extended contour length of approximately 19 nm. The Rg values indicate that all polymers remain in highly coiled conformations, allowing direct comparison across architectures.

**Figure 5:**
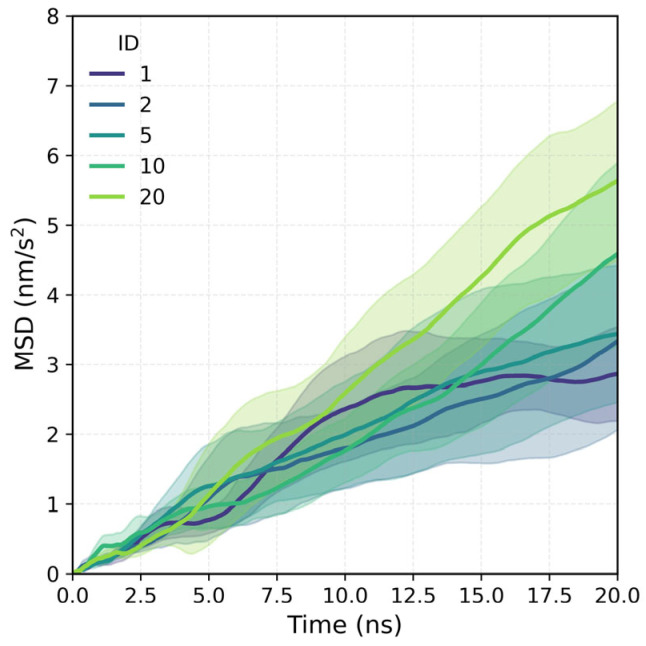
Mean square displacement (MSD) as a function of time for polymers with different ID architectures in aqueous solution.

Figure 5 shows the mean square shift (MSD); a clear trend of increasing molecular mobility is observed as the polymer presents longer charged blocks. Systems with homogeneous load distribution (cases 1 and 2) have lower MSD values, which reflects a more restricted and coherent dynamic with their more widespread conformations. In contrast, polymers with extensive loaded blocks (cases 10 and 20) show a sustained increase in MSD, indicating greater freedom of movement and faster diffusion into the aqueous medium. This trend is consistent with the results of the turning radius: as the polymer adopts a more compact conformation, the neutral chain of PAM becomes dominant in the overall behavior and is less affected by interactions with water or electrostatic effects. In the absence of ions, mobility is mainly determined by the neutral fraction, which exhibits less friction with the solvent. These results show that the load distribution modifies the static conformation of the polymer and its translational dynamics, which indicates that the more blocked architectures favor greater mobility in pure water.

**Figure 6:**
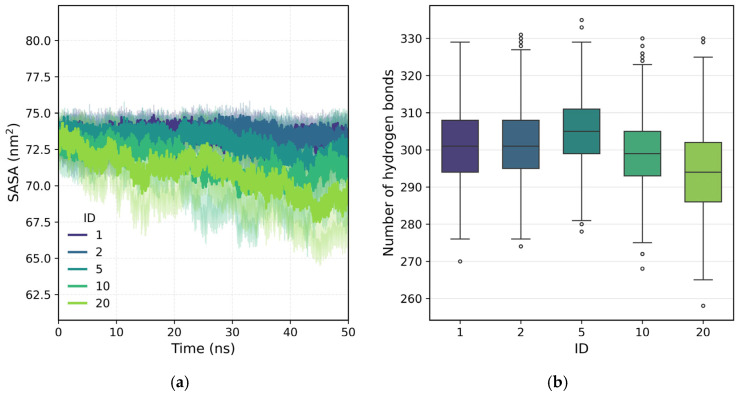
(**a**) Time evolution of the solvent-accessible surface area (SASA) for polymers with different ID architectures in aqueous solution. (**b**) Polymer–water hydrogen bond box distributions for different ID architectures.

Figure 6a shows the temporal evolution of the solvent accessible surface area (SASA); the results show a progressive decrease in SASA as the chain presents more extensive loaded blocks, in accordance with the observed reduction in the turning radius. Polymers with homogeneous charge distribution (cases 1 and 2) maintain SASA values close to 74–75 nm^2^, indicating structures that are more exposed to the solvent. In contrast, systems with larger load blocks (10 and 20) have values that drop to ~68–70 nm^2^, reflecting more compact conformations in which a significant portion of the neutral chain is less accessible to water. This behavior confirms that the structural compaction induced by the redistribution of loads affects the overall geometry of the polymer and its interaction with the solvent. In the absence of ions, the lower exposure of the neutral segments suggests a more internal organization of the chain, in which the electrostatic repulsions located in the charged blocks determine the degree of folding and surface accessibility. This interpretation is further supported by the analysis of hydrogen bonding (Figure 6b), which shows that polymers with larger ID exhibit a lower average number of polymer–water hydrogen bonds. The reduction in hydrogen bonding is consistent with the decrease in SASA, confirming that charge segregation promotes more compact conformations in which neutral segments are less exposed to the solvent.

### 3.2. Asorption in Quartz Surface

**Figure 7:**
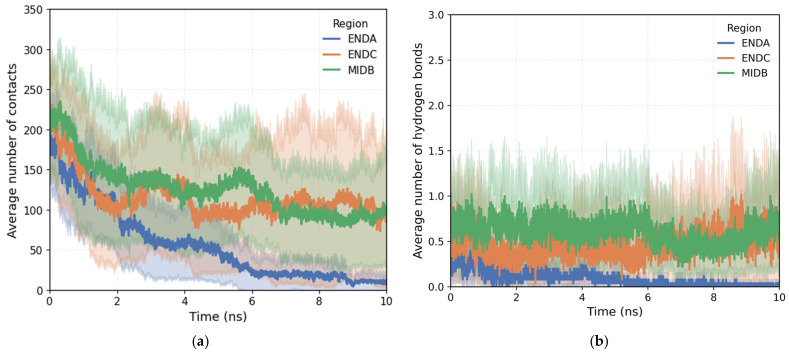
Time evolution of the average number of polymer–quartz contacts (**a**) and hydrogen bonds (**b**) for the ENDA, MIDB, and ENDC initial configurations.

After analyzing the conformation of the different polymers in solution, the stability of their adsorption onto the quartz surface in pure water was evaluated. The analysis presented here represents an average over all polymer architectures investigated (ID = 1–20, Table 1) and is intended to highlight general trends associated with the initial adsorption configuration. Figure 7a shows the time evolution of the average number of contacts for the three initial configurations studied: ENDA, corresponding to the end where the charged carboxylate groups are concentrated; MIDB, which positions a central region of the polymer; and ENDC, where the acrylamide end is oriented toward the mineral. Initially, the three systems exhibit a comparable number of contacts (≈170–250), reflecting similar starting conditions despite the differences in the initial polymer orientation. As the simulation progresses, a general decrease in the number of contacts is observed, associated with conformational reorganization processes and partial desorption events. However, the ENDC and MIDB configurations show more stable adsorption over time, maintaining significantly higher contact values than those of ENDA. This behavior suggests that adsorption on quartz is favored when the surface interacts preferentially with neutral segments of the polymer, whether concentrated at the end (ENDC) or in the central region (MIDB). In contrast, ENDA, dominated by charged groups, exhibits a more pronounced progressive loss of contacts, which is consistent with the lower affinity of quartz for charged monomers reported in previous studies [41]. The analysis of hydrogen bonding between polymer and quartz surface (Figure 7b) reveals a similar trend. Although the absolute number of hydrogen bonds is relatively low, MIDB and ENDC maintain consistently higher hydrogen-bond populations than ENDA throughout the simulation. This indicates that hydrogen bonding contributes to adsorption stability primarily when neutral acrylamide-rich regions are exposed to the surface, while configurations dominated by charged groups rely less on specific hydrogen-bond interactions and are more prone to desorption. It should be noted that the contact curves represent averages over multiple simulations. Therefore, a decrease in the average number of contacts toward zero reflects desorption events in a fraction of the trajectories, with polymer chains moving away from the surface into the bulk solution.

**Figure 8:**
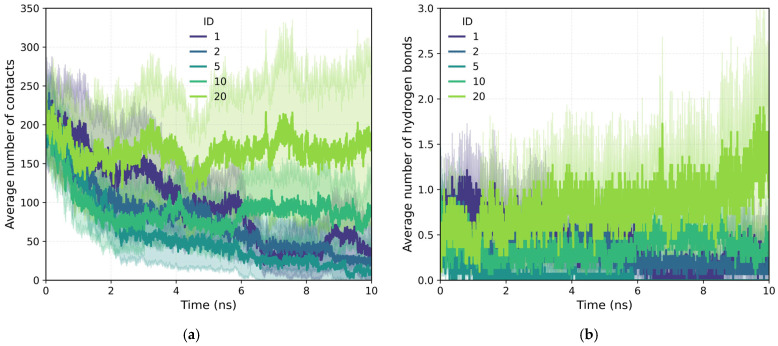
Time evolution of the average number of polymer–quartz contacts (**a**) and hydrogen bonds (**b**) for the different ID configurations.

In addition to evaluating the influence of the initial adsorption configuration, the effect of the polymer’s ID architecture on the stability of surface contacts was analyzed. Figure 8a shows the time evolution of the average number of contacts between polymers with different sizes of loaded blocks (ID = 1–20), together with the corresponding evolution of polymer–quartz hydrogen bonds (Figure 8b). In all cases, an initial decrease in contacts is observed in the first few nanoseconds, associated with conformational reorganization processes and partial desorption from the surface.

However, clear differences are observed depending on the size of the loading block. Polymers with large blocks of 10 and, especially, 20 monomers show significantly more stable adsorption, maintaining higher contact values throughout the simulation. The case of 20 monomers stands out consistently, with a high number of contacts that even increases after the initial reorganization phase, indicating robust and persistent adsorption.

In contrast, architecture with smaller blocks (1, 2, and 5 monomers) exhibit a progressive loss of contacts and greater fluctuations, reflecting a lower effective affinity with the surface and a higher propensity for desorption. Polymers with high ID, particularly ID = 20, display not only more persistent surface contacts but also a higher and more stable number of polymer–surface hydrogen bonds (Figure 7b), which reinforces their adsorption stability.

This behavior suggests that greater intramolecular segregation between charged and neutral regions favors stable adsorption on quartz, allowing longer neutral segments to establish preferential contact zones with the surface, while the charged regions remain mostly exposed to the solvent. Taken together, these results reinforce the idea that architectural heterogeneity and the presence of long neutral blocks—particularly in the case of 20 monomers—are key factors in enhancing adsorption stability, consistent with the trends observed for the ENDC configuration.

To complement the quantitative contact analysis (Figure 7), representative simulation snapshots were examined to illustrate polymer rearrangement at the quartz surface (Table 2). The initial configurations correspond to the final stage of the steered molecular dynamics (SMD) protocol, which induces relatively flattened, surface-oriented conformations that are expected to favor adsorption upon equilibration. Despite these favorable starting configurations, polymers with homogeneous charge distributions (ID = 1) exhibit more extended conformations with intermittent surface contacts and partial desorption, indicating limited adsorption stability. In contrast, block-like architectures with long neutral segments (ID = 20) form more compact conformations, where neutral PAM segments remain stably adsorbed while charged blocks preferentially extend into the aqueous phase. This behavior is consistent with the higher and more stable number of contacts observed for large block architectures in Figure 7.

**Figure 9:**
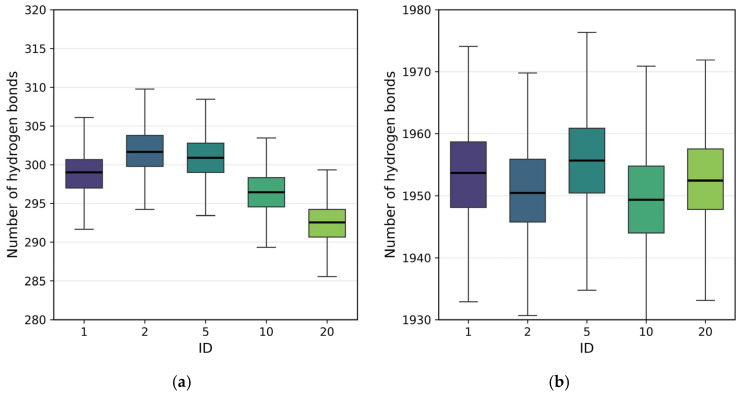
Distribution of hydrogen bonds during the adsorption–desorption process for polymers with different ID architectures: (**a**) polymer–water hydrogen bonds and (**b**) surface–water hydrogen bonds.

As a final analysis, Figure 9 presents the hydrogen bond distributions during the adsorption–desorption process, distinguishing polymer–water (Figure 9a) and surface–water interactions (Figure 9b). Polymer–water hydrogen bonding shows a weak but clear dependence on the charge-site architecture. Polymers with intermediate block sizes (ID = 2 and 5) display slightly higher average numbers of hydrogen bonds, consistent with the non-monotonic trend previously observed for SASA (Figure 6b). This behavior suggests the existence of an optimal architecture that enhances polymer hydration, indicating that moderate charge segregation may improve polymer–water interactions without inducing strong chain compaction. In contrast, the number of surface–water hydrogen bonds remain nearly constant for all IDs (Figure 9b), reflecting the large number of interfacial water molecules and the resulting dilution of architecture-dependent effects. Nevertheless, direct polymer–surface hydrogen bonds (Figure 7b and Figure 8b) show clear and reproducible trends, confirming the formation of stable, architecture-dependent interfacial interactions.

## 4. Discussion

The molecular trends identified in this work are consistent with experimental studies reporting that polyacrylamide adsorption on mineral surfaces is governed primarily by polymer conformation and segment accessibility rather than by molecular weight or global charge density alone [47,48]. Experimental adsorption studies have shown that partially compacted chains can establish more stable surface contacts than fully extended conformations.

Direct experimental evidence further supports this interpretation. Atomic force microscopy and adsorption measurements have revealed heterogeneous surface coverage of polyacrylamide on mineral substrates, where stable attachment arises from localized anchoring segments rather than uniform chain adsorption [49,50]. These observations are consistent with the present results, which show that charge-site distribution promotes compact conformations with defined surface-contact regions.

Additional experimental studies have highlighted the role of hydrogen bonding and local polymer–surface interactions in stabilizing adsorption, particularly for amide-containing polymers interacting with hydroxylated mineral surfaces [51,52].

In agreement with these findings, the present results show that architectures containing long neutral blocks, particularly ID = 20, form a higher and more persistent number of polymer–surface hydrogen bonds, which contributes directly to their enhanced adsorption stability on quartz. In contrast, polymers with more homogeneous charge distributions exhibit fewer and more transient hydrogen-bond interactions with the surface, leading to weaker anchoring and increased desorption.

Together, these findings support the conclusion that intramolecular charge distribution constitutes a key structural parameter controlling polymer conformation, adsorption stability, and ultimately flocculation performance.

## 5. Conclusions

In this work, the effect of the charge architecture on anionic polyacrylamides was investigated using classical molecular dynamics, ranging from polymers with homogeneous distribution of charged groups to charged-neutral block copolymer architectures. The results show that the intramolecular redistribution of the fillers induces significant changes in the conformation and dynamics of the chains, affecting their degree of stretching, their diffusion capacity and their exposure to the solvent. As the polymer evolves towards a block architecture, a decrease in the turning radius is observed, accompanied by an increase in translational mobility in solution and a reduction in solvent accessibility.

These behaviors are explained by the spatial segregation between charged and neutral segments, which favors the formation of heterogeneous structures dominated by the majority monomers, in this case of a neutral character (≈75%). This segregation promotes intramolecular folding of the neutral backbone and limits solvent exposure under low-ionic-strength conditions. In adsorption analysis, architectures that concentrate neutral segments in large blocks exhibit more stable adsorption on the quartz surface, in agreement with previous studies that indicate a greater affinity of this surface—characterized by a low charge density—towards neutral or weakly charged polymers. This enhanced stability is directly associated with the formation of persistent polymer–surface hydrogen bonds involving neutral acrylamide segments.

These results demonstrate that charging architecture constitutes a key parameter to modulate both the properties in solution and the stability of the interfacial adsorption of anionic polyacrylamides. Importantly, charge-site architecture emerges as an independent design variable beyond molecular weight or global charge density. The presented approach opens the possibility of designing optimized flocculants for applications in mine tailings and other particulate systems, through rational control of the sequence and distribution of monomers.

## Figures and Tables

**Table 1:** Polyacrylamide configurations, A = CH_2_–CH(COO^−^), B = CH_2_–CH(CONH_2_).

ID	N Block Monomer Total	N Block Monomer Charged	N Block Monomer Neutral	Notation
1	4	1	3	H–[A_1_B_3_]_20_–H
2	8	2	6	H–[A_2_B_6_]_10_–H
5	20	5	15	H–[A_5_B_15_]_4_–H
10	40	10	30	H–[A_10_B_30_]_2_–H
20	80	20	60	H–[A_20_B_60_]_1_–H

**Table 2:** Representative initial (left) and final (right) configurations from the production MD stage for selected systems. Neutral PAM segments are shown in blue, charged segments in red. Negatively charged surface sites are highlighted in green.

ID	ENDA	MIDB	ENDC
1	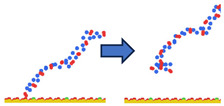	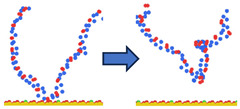	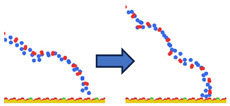
20	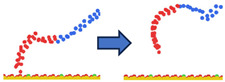	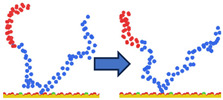	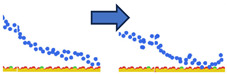

## Data Availability

The raw data supporting the conclusions of this article will be made available by the authors on request.

## Supplementary Materials

The following supporting information can be downloaded at: https://www.mdpi.com/article/10.3390/polym18030414/s1, The folders contain all input files required to run the molecular dynamics simulations in Gromacs for Systems S1 and S2 at ID = 1 and ID = 20.

## Abbreviations

The following abbreviations are used in this manuscript:

HPAM	Hydrolyzed polyacrylamide
SMD	Steered Molecular Dynamics
GAFF	Generar Amber Force Field
CLAYFF	Clays Force Field
IOD	Ion Oxygen Distance
DLVO	Derjaguin–Landau–Verwey–Overbeek theory
MSD	Mean Square Displacement
SASA	Solvent Accessible Surface Area
